# The Hopkins-Hammond Controversy

**Published:** 1884-12

**Authors:** J. G. Hopkins

**Affiliations:** Thomasville, Ga.


### THE HOPKINS-HAMMOND CONTROVERSY.

THOMASVILLE, GA., Nov. 12th, 1884.

*Editors Atlanta Medical and Surgical Journal:*

In the letter of Dr. W. A. Hammond of October 10th, in reply to mine, which appeared in your Journal of that month, I find such utter abandonment of honesty and truth that I am more fully than ever convinced of the correctness of my act in criticising him as I did. He deals in sophistical sarcasm, denies my assertions, and makes for himself others which are as wholly untrue as the tales connected with the mythologic ornations of his man-trap. Even if his accusations were undeniable facts they could not stamp me with that infamy which to-day overshadows the Doctor himself. I have never been publicly charged in a medical journal with having accepted a bribe of twenty-five hundred dollars to reverse my opinion in an important will case. He has, as will be seen from the following by Dr. Grisom, taken from the Philadelphia Times of Nov. 9th, 1868:

"First. In order to clear McFarland, who shot Richardson, Dr. Hammond stated on the witness stand, 'that the insane are very persistent in their revenge. I have known insane men occupied with the idea of killing their keeper for years, and finally do it;' while, in order to convict Montgomery, he stated 'deliberation takes away the idea of an insane act."

"Second. That to insure the execution of Reynolds, Dr. Hammond declared under oath, 'the disease (epileptic mania) is of remarkably short duration. There is not a case on record where it has lasted fifteen minutes;' whilst in order to convict Montgomery he said, 'when an epileptic has suffered from an attack, the mental disturbance continues frequently several days.' "

"Third. That in the Johnston will case he gave testimony which was scientifically false, although necessary for the breaking of the will, and that it was proved that he was to receive $500 for his testimony, and a contingent of $2,500 if he succeeded in breaking the will.

"Fourth. That in the Montgomery case he one day gave one opinion, and the following day an opposite one, having been seen in the meanwhile by the interested counsel."

These charges have not only been publicly preferred against Dr. Hammond by Dr. Grisom, who is a member of the judicial council of the American Medical Association, but they have been sustained by that council and the National Association of Specialists connected with the subject. Dr. Hammond replied to these charges in a voluminous outburst of foul expressions, exhaustive of the English vocabulary, and highly characteristic of the blackguard that he is.

If he can find honor in being "fired back again" into the United States Army, and placed upon the retired list without pay, after having been cashiered for rascality and kicked out, I fail to see it. I challenge him to produce evidence of one single instance in my life wherein I proved unfaithful to a trust. If I have slandered him he has his remedy. Why has he not recourse to it? Not so; his guilty conscience (if he has any) has precluded it.

I have owned and used the ophthalmoscope—my knowledge of which he doubts—during the ten years I have been a practitioner of medicine, and I am aware that the differential calorimeter is owned by others, but these "little instruments" are exercised in their proper sphere and not applied to nefarious schemes, as is this unfortunate one in the possession of Dr. Hammond.

As to my "fingering" his funds, this was not necessary; he saves every one this trouble, for they are carelessly (?) thrown together so that the figures, 20 can be seen from almost any quarter of his apartment. On one occasion I called before he finished his breakfast; there was one twenty-dollar bill on the desk, and he remarked, "Almost first this morning, Doctor." His porter had informed me that none but myself and my patient had been there that morning, nor had the Doctor been out. He has no "family practice." On another day, I called at eleven o'clock, and, according to the porter and what I saw for myself, only four patients had presented themselves up to that hour. Three were old patients I had seen several times before; one was uncertain, but when I entered that sanctum sanctorum there were 8 or 10 twenty-dollar bills upon his desk These, also, had been placed with the same *accurate carelessness* as heretofore.

In reply to his demand for an author of the story of the chicken bone operation, I would say, that this intelligence was gained in the private office of a gentleman and not at a circus, and I refuse to comply, and charge him to hold me in person responsible.

Dr. Hammond did examine my urine, at least I gave it to him for that purpose. Corroborating the opinion of others he diagnosed my case one of chronic gastritis, and prescribed accordingly. I only desired his opinion, hence I have not nor will I ever take his medicine. He claims to have diagnosed "serious brain trouble." This condition may exist, but so far as his assertion relative to the examination goes, I am free to assert that it is in keeping with most that he has said—utterly false. Perhaps he judges me by himself. One would readily imagine from his personal appearance that he carried his brain in his belly. He once diagnosed "Serious brain trouble" in a case of Dr. W. F. Westmoreland's, which accidentally fell into his hands—somewhat on the order, I presume, of the accident which nearly got him into the Garfield case. In this instance the brain of the patient must have been carried in his pants, as Dr. Sayre of New York treated him immediately thereafter for hip-joint disease and he rapidly recovered.

Dr. Hammond accuses me of encouraging the boy's "insane penchant." That his family is convinced of the falsity of this is amply sufficient to me. It was through their appeals that I permitted his following. I assured them that this action would but increase his trouble and make bad matters worse. He was a relative from whom I accepted scant remuneration (I was privileged to charge as I pleased) so I could have had no object in encouraging him. I told Dr. Hammond that ten thousand dollars would be no inducement to thus leave my family, my practice and undergo such annoyance; that it was my affection for the family which prompted me; that I had grown very tired of it, however, and often felt like shamming a quarrel and quitting him. His reply was "d—n him, I'd take up a stick and beat him to death."

Some time after we had returned to Atlanta, the patient became convinced that no ill could befall him and he was gradually withdrawn from me. Up to the time of my returning to Thomasville—my old home—there was no improvement except in this particular, he was no better when he wrote to Dr. Hammond.

I wish, in conclusion, to repeat with much emphasis, all that is said above, and all that I have heretofore said, as well as to assert that Dr. Hammond is an unblushing falsifier, and the truth is not in him.

J. G. HOPKINS, M. D.

